# Pattern and risk factors for distant metastases in gastrointestinal neuroendocrine neoplasms: a population‐based study

**DOI:** 10.1002/cam4.1507

**Published:** 2018-05-07

**Authors:** Wen Cai, Yinuo Tan, Weiting Ge, Kefeng Ding, Hanguang Hu

**Affiliations:** ^1^ Department of Medical Oncology the Second Affiliated Hospital School of Medicine Zhejiang University Hangzhou Zhejiang China; ^2^ Cancer Institute (Key Laboratory of Cancer Prevention and Intervention, China National Ministry of Education) the Second Affiliated Hospital School of Medicine Zhejiang University Hangzhou Zhejiang China; ^3^ Department of Surgical Oncology the Second Affiliated Hospital School of Medicine Zhejiang University Hangzhou Zhejiang China

**Keywords:** Gastrointestinal, Metastases, Neuroendocrine neoplasms, Risk factors, SEER

## Abstract

An increased incidence of gastrointestinal neuroendocrine neoplasms (GI‐NENs) has been reported worldwide, and metastasis is the leading cause of GI‐NEN‐related death. Studies of different metastatic patterns in patients with different primary sites are limited. A population‐based retrospective cohort study was conducted with the Surveillance, Epidemiology, and End Results (SEER) database. Patients with a GI‐NEN diagnosis between 2010 and 2014 were included. All statistical analyses were performed using Intercooled Stata 12.0 software. There were 12,501 patients eligible for analysis. The metastatic status, primary sites, and histology types affected the patients’ overall survival. The liver was the most common metastasis site (65.21% of patients with metastases). Esophageal NENs had the highest risk of metastasis (49.35%), whereas appendiceal NENs had the lowest risk of metastasis (2.79%). Neuroendocrine carcinomas (NECs) were more likely to develop metastatic disease than were neuroendocrine tumors (NETs); 7.12% of patients with NET and 30.20% of patients with NEC developed metastatic disease. The metastatic patterns varied according to the different primary sites and histology types. NECs had a higher potential to develop extrahepatic metastasis at all primary sites than did NETs. Regarding the choice of treatment, surgical resection of primary lesions lowered the risk of tumor‐specific death (HR = 0.37, CI: 0.30–0.46, *P *< 0.01), but surgical resection of metastatic sites did not confer an extra survival benefit (HR = 0.82, CI: 0.63–1.06, *P *=* *0.14). Different primary sites and histology types of GI‐NENs have different metastatic patterns and survival. This knowledge could help clinicians to identify patients who require extra surveillance, provide insight for future studies on the mechanisms of metastasis, and establish a prognostic prediction model.

## Introduction

Neuroendocrine neoplasms (NENs) are a diverse group of tumors that arise from neuroendocrine cells throughout the body and have similar features 1. Gastrointestinal neuroendocrine neoplasms (GI‐NENs) are defined as neuroendocrine neoplasms that arise in the gastrointestinal tract 2, 3. Tumors of this subtype frequently grow slowly and behave as chronic oncological diseases with a relatively long survival. The most recent 2010 WHO system has defined all neuroendocrine tumors (NETs) as neoplasms with malignant potential, and the acronym NEN is recommended to corresponding to the term neuroendocrine neoplasia. NENs are currently classified as either well‐differentiated (low‐grade to intermediate‐grade) neuroendocrine tumors (NET) or poorly differentiated (high‐grade) neuroendocrine carcinoma (NEC) based on the morphology and proliferation rate 4, 5. It is generally acknowledged that survival is better for NET than for NEC. However, previous studies have often focused on only NET or NEC, and thus, it is difficult to describe survival differences between NET and NEC precisely.

The annual age‐adjusted incidence of NENs was 1.09 per 100,000 persons in 1973 and increased to 6.98 per 100,000 persons by 2012 6. The epidemiology of different NEN subsets has been well‐studied; however, studies focusing on metastasis events in GI‐NENs have been limited 7.

As is commonly known, the metastatic spread of cancer to distant organs is the primary cause of most cancer‐related deaths. However, most NENs are clinically silent until the advanced stages of disease such as the detection of metastatic lesions. Although these lesions are generally more indolent than carcinomas, they often have unpredictable biological behavior and are on occasion associated with a very aggressive clinical course. Unfortunately, there are limited data and studies describing the epidemiology and survival of individuals with metastatic GI‐NENs. The 5‐year survival rate for patients with poorly differentiated NEC varies between 6% and 11% depending on the European region 8. No articles have reported big data on metastatic GI‐NET survival or survival differences between GI‐NET and GI‐NEC.

We conducted this study with the Surveillance, Epidemiology, and End Results (SEER) database to describe the metastatic spectrum and survival in primary site‐specific metastases from NET and NEC and to explore the risk factors for metastases in the GI tract. We aim to provide more evidence for physicians regarding the knowledge of different metastatic spectra from different primary sites and risk factors for metastatic disease, which may help guide pretreatment evaluations of patients and detect the probable primary sites for metastatic NENs. The further survival analysis is intended to help physicians make decisions regarding optimal follow‐up strategies.

## Methods

This study utilized data from the US SEER database. The SEER*Stat software program (version 8.3.4) was used to extract data on neuroendocrine neoplasm patients from the database, which includes patients between 1973 and 2014 (November 2016 sub). Information regarding specific metastasis sites, including metastases to the bone, brain, liver, lung, and distant lymph nodes, was recorded beginning in the year 2010, and thus, we extracted these data from 2010 to 2014. ICD‐O‐3 histology codes were used to identify primary sites, including the esophagus, stomach, small intestine, appendix, colon, and rectum. These codes correspond to the following clinical/histologic diagnoses: neuroendocrine neoplasms (8240), NET (8249), gastrinoma (8153), somatostatinoma (8156), endocrine tumor (8158), gangliocytic paraganglioma (8683), and neuroendocrine carcinoma (8246). We excluded cases if (1) the survival data description was incomplete; (2) the description of the metastatic status was incomplete; or (3) there were multiple primary tumors.

The patients’ demographic and tumor characteristics were summarized with descriptive statistics. Comparisons of categorical variables among the different groups of patients were performed using the chi‐square test. Deaths attributed to NENs were treated as events. Univariate Cox regression was performed to identify risk factors of survival, and further multivariate Cox regression was performed to select independent prognostic risk factors. Survival function estimation and comparisons among different variables were performed using Kaplan–Meier estimates and Cox regression analyses. Univariate and multivariate logistic regression analyses were performed to identify variables that might influence the metastatic status. All the statistical analyses were performed using Intercooled Stata 12.0 (Stata Corporation, College Station, TX). Statistical significance was set when the two‐sided *P* value <0.05.

## Results

### Epidemiological characteristics of the included patients

**Figure 1:**
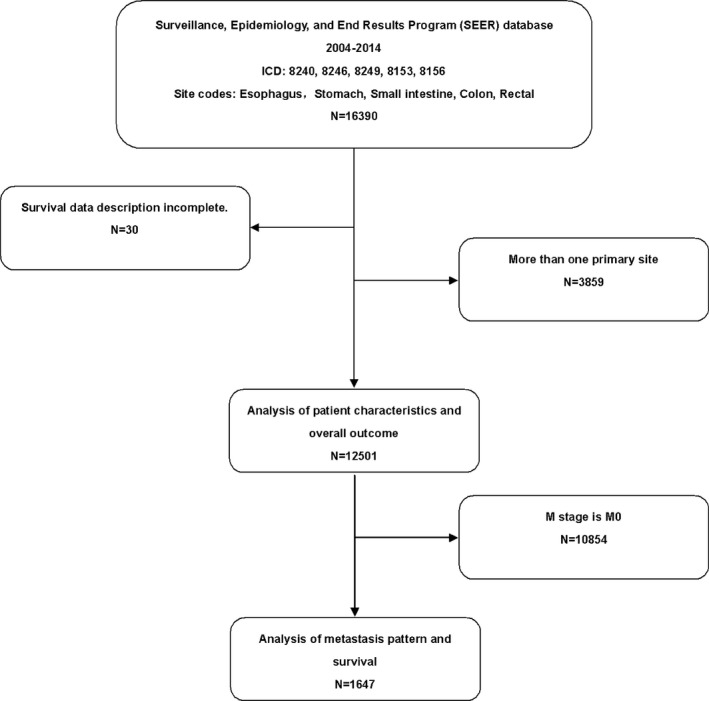
Flow chart depicting the selection of patients.

A total of 16,390 patients with GI‐NENs who were diagnosed from 2010 to 2014 were identified from the SEER database. After eliminating 30 cases with incomplete survival data and 3859 cases with multiple primary sites, 12,501 patients were included for further analysis (Fig. 1). NENs were found throughout the GI tract. The most frequent primary sites were the rectum (35.54%) and small intestine (33.64%). Esophageal NENs (0.62%) and stomach NENs (1.88%) were rare. GI‐NETs represented the main proportion at each primary site except the esophagus, for which NEC accounted for 94.81%. The proportion of NET and NEC at each primary site and the patients’ detailed basic characteristics are presented in Table 1. Among the entire cohort, 1647 patients (13.17%) developed metastatic disease. NENs originating from the esophagus had the highest metastasis rate (49.35%) with metastases originating from the small intestine (28.71%) and colon (26.08%) also showing a high frequency. Appendicular NENs showed the lowest rate (2.79%) of metastatic disease. Although the rectum was the most common primary site, only 2.99% of patients with this subtype developed distant metastasis.

**Table 1:** Clinical features and primary sites

Features	Esophagus	Stomach	Duodenum	Jejunum and Ileum	Appendix	Colon	Rectum	Unspecific primary site	*P* value
Histology (%)									
NET	4 (5.19)	110 (69.62)	1651 (71.44)	1218 (64.31)	599 (69.73)	383 (57.08)	3792 (85.35)	1462 (70.02)	<0.01
NEC	73 (94.81)	48 (30.38)	660 (28.56)	676 (35.69)	260 (30.27)	288 (42.92)	651 (14.65)	626 (29.98)	
Gender (%)									
Female	13 (16.88)	104 (65.82)	1155 (49.98)	948 (50.05)	535 (62.28)	334 (49.78)	2232 (50.24)	1248 (59.77)	<0.01
Age (%)									
<60 year‐ old	14 (18.18)	70 (44.30)	806 (34.88)	893 (47.15)	732 (85.22)	346 (51.56)	3026 (68.11)	1004 (48.08)	<0.01
Race (%)									
White	66 (85.71)	120 (75.95)	1665 (72.05)	1632 (86.17)	718 (83.59)	460 (68.55)	2283 (51.38)	1580 (75.67)	<0.01
Insurance (%)									
Insurance	50 (64.94)	83 (52.53)	1554 (67.24)	1400 (73.92)	510 (59.37)	378 (56.33)	2622 (59.01)	1239 (59.34)	<0.01
Marital status (%)									
Married	43 (55.84)	75 (47.47)	1249 (54.05)	1159 (61.19)	348 (40.51)	314 (46.80)	2225 (50.08)	1076 (51.53)	<0.01
Grade (%)									
I	3 (3.90)	76 (48.10)	1242 (53.74)	1249 (65.95)	600 (69.85)	209 (31.15)	1900 (42.76)	909 (43.53)	<0.01
II	0 (0.00)	7 (4.43)	241 (10.43)	291 (15.36)	104 (12.11)	51 (7.60)	325 (7.31)	227 (10.87)	
III	52 (67.53)	15 (9.49)	32 (1.38)	24 (1.27)	7 (0.81)	120 (17.88)	87 (1.96)	139 (6.66)	
IV	12 (15.58)	3 (1.90)	8 (0.35)	5 (0.26)	7 (0.81)	40 (5.96)	32 (0.72)	64 (3.07)	
Unspecific	10 (12.99)	57 (36.08)	788 (34.10)	325 (17.16)	141 (16.41)	251 (37.41)	2099 (47.24)	749 (35.87)	
Tumor size (%)									
<1 cm	1 (1.30)	46 (29.11)	734 (31.76)	335 (17.69)	477 (55.53)	167 (24.89)	2241 (50.44)	653 (31.27)	<0.01
1–2 cm	2 (2.60)	19 (12.03)	572 (24.75)	725 (38.28)	205 (23.86)	32 (4.77)	204 (4.59)	222 (10.63)	
2–3 cm	3 (3.90)	10 (6.33)	249 (10.77)	446 (23.55)	68 (7.92)	28 (4.17)	69 (1.55)	169 (8.09)	
3–4 cm	7 (9.09)	2 (1.27)	83 (3.59)	147 (7.76)	29 (3.38)	25 (3.73)	40 (0.90)	117 (5.60)	
4–5 cm	3 (3.90)	3 (1.90)	52 (2.25)	53 (2.80)	14 (1.63)	38 (5.66)	35 (0.79)	61 (2.92)	
>5 cm	27 (35.06)	9 (5.70)	79 (3.42)	50 (2.64)	16 (1.86)	93 (13.86)	60 (1.35)	135 (6.47)	
Unspecific	34 (44.16)	69 (43.67)	542 (23.45)	138 (7.29)	50 (5.82)	288 (42.92)	1794 (40.38)	731 (35.01)	
T‐stage (%)									
T2	6 (7.79)	27 (17.09)	355 (15.36)	385 (20.33)	90 (10.48)	32 (4.77)	191 (4.30)	275 (13.17)	<0.01
T3	11 (14.29)	10 (6.33)	577 (24.97)	785 (41.45)	30 (3.49)	114 (16.99)	71 (1.60)	284 (13.60)	
T4	11 (14.29)	9 (5.70)	278 (12.03)	442 (23.34)	20 (2.33)	65 (9.69)	27 (0.61)	157 (7.52)	
Unspecific	49 (63.64)	112 (70.89)	1101 (47.64)	282 (14.89)	719 (83.70)	460 (68.55)	4154 (93.50)	1372 (65.71)	
N‐stage (%)									
N0	20 (25.97)	115 (72.78)	1358 (58.76)	511 (26.98)	716 (83.35)	386 (57.53)	3675 (82.71)	1339 (64.13)	<0.01
N1	37 (48.05)	17 (10.76)	753 (32.58)	1345 (71.01)	111 (12.92)	178 (26.53)	123 (2.77)	457 (21.89)	
N2	5 (6.49)	0 (0.00)	1 (0.04)	0 (0.00)	0 (0.00)	0 (0.00)	0 (0.00)	0 (0.00)	
Unspecific	14 (18.18)	26 (16.46)	199 (8.61)	38 (2.01)	32 (3.73)	107 (15.95)	645 (14.52)	292 (13.98)	
M‐stage (%)									
M0	39 (50.65)	141 (89.24)	1882 (81.44)	1385 (73.13)	835 (97.21)	496 (73.92)	4310 (97.01)	1766 (84.58)	<0.01
M1	38 (49.35)	17 (10.76)	429 (18.56)	509 (26.87)	24 (2.79)	175 (26.08)	133 (2.99)	322 (15.42)	
Primary site surgery (%)									
Yes	8 (10.39)	82 (51.90)	1730 (74.86)	1788 (94.40)	835 (97.21)	446 (66.47)	3520 (79.23)	1378 (66.00)	<0.01
No	69 (89.61)	76 (48.10)	581 (25.14)	106 (5.60)	24 (2.79)	225 (33.53)	923 (20.77)	710 (34.00)	
Metastasis site surgery (%)									
Yes	2 (2.60)	12 (7.59)	211 (9.13)	301 (15.89)	56 (6.52)	39 (5.81)	138 (3.11)	102 (4.89)	<0.01
No	75 (97.40)	146 (92.41)	2100 (90.87)	1593 (84.11)	803 (93.48)	632 (94.19)	4305 (96.89)	1986 (95.11)	

### Exploring risk factors and survival analysis of the included patients

We performed univariate Cox regression first to identify variables that might influence survival. We then selected independent prognostic risk factors using multivariate Cox regression. Race, age, tumor grade, tumor sizes, primary site of tumor, TNM stage, NET or NEC, and surgery performed at the primary site and metastatic site were identified as independent prognostic risk factors for GI‐NEN survival. Furthermore, we divided the factors into subgroups and chose one of them as the reference to identify the specific factors. The results presented in Table 2 suggest that age older than 60, a lack of insurance, a tumor grade higher than III, tumor size larger than 3 cm in diameter, T4 stage, N1 stage, developing metastatic disease, diagnosis as NEC, and different primary sites independently increase the risk of tumor‐specific death. When we used esophageal NENs as a reference and adjusted the influence of gender, race, and marital status, we observed that primary NENs from the stomach (HR = 0.49, CI: 1.24–0.99, *P *=* *0.05) and colon (HR = 0.94, CI: 0.66–1.34, *P *=* *0.74) showed no significant difference in survival. By contrast, the other primary sites all showed better survival than esophageal NENs. We took all risk factors selected from the multivariate analysis into consideration, which suggested that surgical resection of the primary sites could lower the risk of tumor‐specific death (HR = 0.37, CI: 0.30–0.46, *P *< 0.01), whereas surgical resection of the metastatic sites did not confer an extra survival benefit (HR = 0.82, CI: 0.63–1.06, *P *=* *0.14).

**Table 2:** Univariate and multivariate analyses of GI‐NENs‐specific survival

Variable	Univariate analyses		Multivariate analyses	
	Hazard ratio (95% CI)	*P* value	Hazard ratio (95% CI)	*P* value
Gender				
Female	1.00		1.00	
Male	1.26 (1.09–1.46)	<0.01	0.99 (0.85–1.17)	0.95
Race				
White	1.00		1.00	
Black	0.59 (0.48–0.74)	<0.01	0.89 (0.70–1.12)	0.31
Other	0.46 (0.32–0.66)	<0.01	0.78 (0.54–1.12)	0.18
Unspecific	0.09 (0.03–0.28)	<0.01	0.13 (0.04–0.42)	<0.01
Age				
≥60 year‐old	1.00		1.00	
<60 year‐old	2.56 (2.18–3.00)	<0.01	1.76 (1.49–2.08)	<0.01
Insurance				
Insurance	1.00		1.00	
No‐insurance	1.42 (0.96–2.09)	0.08	1.54 (1.03–2.31)	0.03
Unspecific	1.00 (0.85–1.17)	0.99	1.12 (0.94–1.33)	0.20
Marital status				
Married	1.00		1.00	
Unmarried	1.00 (0.82–1.24)	0.96	1.15 (0.92–1.43)	0.22
Unspecific	1.13 (0.95–1.33)	0.17	1.29 (1.08–1.55)	0.01
Grade				
I	1.00		1.00	
II	1.85 (1.33–2.56)	<0.01	1.29 (0.93–1.80)	0.13
III	36.83 (29.58–45.86)	<0.01	4.87 (3.69–6.43)	<0.01
IV	42.66 (32.55–55.92)	<0.01	5.33 (3.87–7.35)	<0.01
Unspecific	1.82 (1.46–2.27)	<0.01	1.80 (1.41–2.30)	<0.01
Tumor size				
<1 cm	0.16 (0.10–0.25)	<0.01	0.47 (0.28–0.77)	<0.01
1–2 cm	1.00		1.00	
2–3 cm	2.13 (1.53–2.96)	<0.01	1.16 (0.83–1.63)	0.38
3–4 cm	4.48 (3.17–6.32)	<0.01	1.46 (1.02–2.10)	0.04
4–5 cm	7.01 (4.91–10.00)	<0.01	1.74 (1.20–2.53)	<0.01
>5 cm	15.53 (11.69–20.64)	<0.01	1.69 (1.23–2.34)	<0.01
Unspecific	2.03 (1.54–2.67)	<0.01	1.20 (0.86–1.68)	0.28
Primary site				
Esophagus	1.00		1.00	
Stomach	0.06 (0.03–0.12)	<0.01	0.49 (0.24–0.99)	0.05
Duodenum	0.04 (0.03–0.06)	<0.01	0.41 (0.27–0.60)	<0.01
Jejunum and Ileum	0.03 (0.03–0.05)	<0.01	0.31 (0.20–0.48)	<0.01
Appendix	0.01 (0.00–0.02)	<0.01	0.20 (0.08–0.49)	<0.01
Colon	0.20 (0.14–0.28)	<0.01	0.94 (0.66–1.34)	0.74
Rectal	0.02 (0.01–0.02)	<0.01	0.36 (0.25–0.53)	<0.01
Unspecific	0.08 (0.06–0.11)	<0.01	0.53 (0.38–0.76)	<0.01
T‐stage				
T2	1.00		1.00	
T3	1.52 (1.17–1.97)	<0.01	1.21 (0.91–1.62)	0.19
T4	3.05 (2.34–3.97)	<0.01	1.45 (1.09–1.93)	0.01
Unspecific	0.54 (0.42–0.69)	<0.01	0.85 (0.63–1.15)	0.30
N‐stage		<0.01		
N0	1.00	<0.01	1.00	
N1	5.17 (4.35–6.15)	<0.01	1.53 (1.26–1.87)	<0.01
N2	9.71 (1.36–69.33)	0.02	0.56 (0.08–4.10)	0.57
Unspecific	4.09 (3.25–5.13)	<0.01	1.27 (0.99–1.62)	0.06
M‐stage				
M0	1.00		1.00	
M1	19.34 (16.42–22.78)	<0.01	5.73 (4.70–6.98)	<0.01
Histology				
NET	1.00		1.00	
NEC	8.95 (7.53–10.64)	<0.01	2.14 (1.73–2.65)	<0.01
Primary site surgery				
No	1.00		1.00	
Yes	0.18 (0.16–0.21)	<0.01	0.37 (0.30–0.46)	<0.01
Metastasis site surgery				
No	1.00		1.00	
Yes	1.63 (1.28–2.08)	<0.01	0.82 (0.63–1.06)	0.14

**Figure 2:**
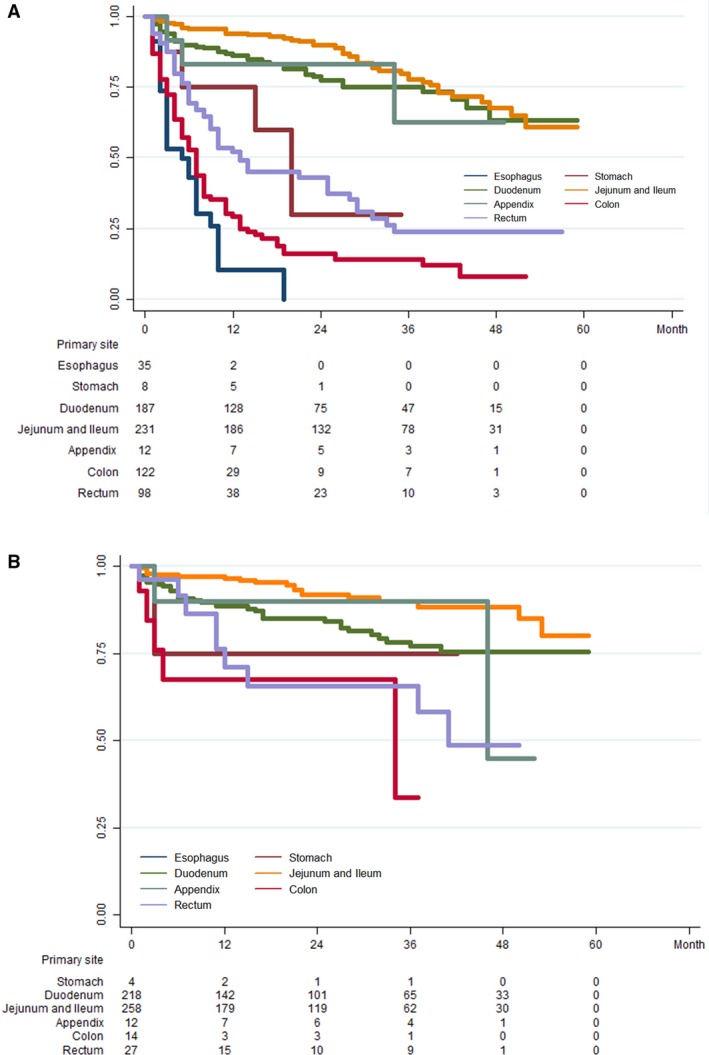
(A) Survival analysis of metastatic NECs in different primary sites. (B) Survival analysis of metastatic NETs in different primary sites.

**Figure 3:**
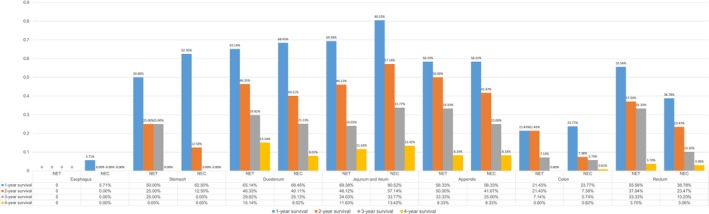
Year specific survival of GI‐NENs at different primary sites.

The survival differences in metastatic disease associated with primary sites are illustrated in Figure 2A and B based on Kaplan–Meier estimates for patients divided into the metastatic GI‐NET group and metastatic GI‐NEC group. We estimated the overall survival (OS) for analysis in the current study, and the 1‐, 2‐, 3‐, and 4‐year OS rates are summarized in Figure 3. We could easily find that only 5.71% patients with esophageal NECs alive in the first year, while none of them survive in the second year. Except NENs originated from jejunum and ileum, NETs shared the better year specific overall survival of each primary sites than NECs. It is very interesting that NECs in jejunum and ileum showed better survival than NETs.

### Metastasis pattern of included patients

**Figure 4:**
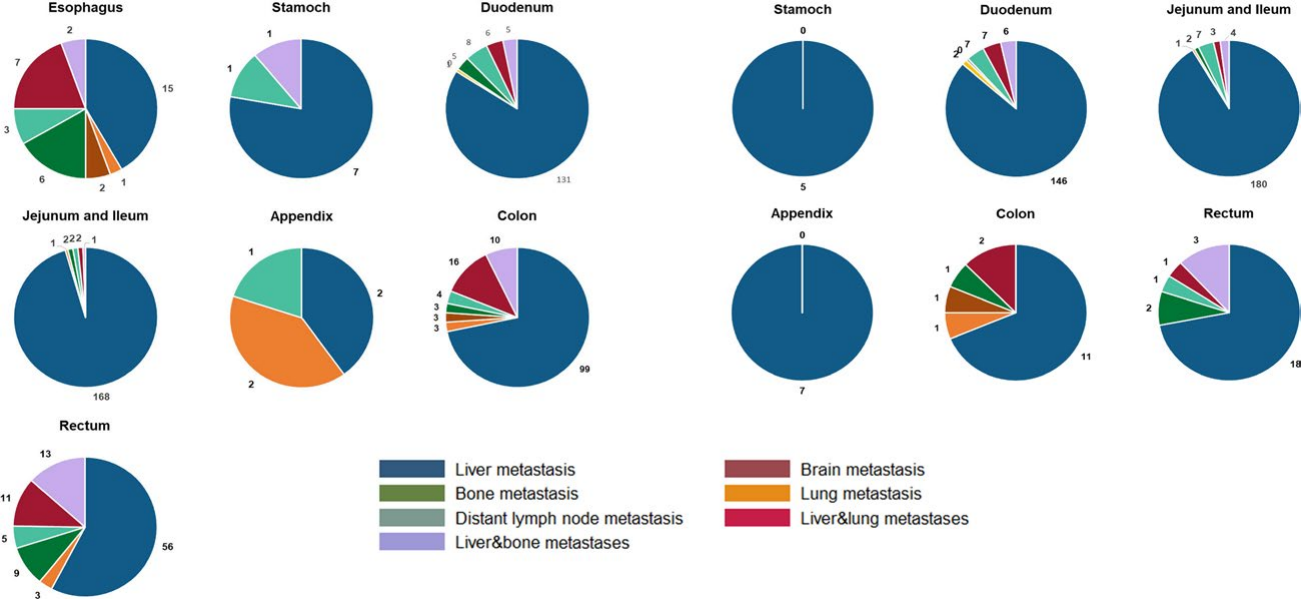
Metastasis pattern of NECs and NETs separated by primary site.

Among 1647 patients who developed metastasis disease, 1162 had a specific record of single organ metastasis (including the liver, lung, brain, bone, and dilatant lymph node). There were 326 patients with M1 disease without a description of the metastatic site, and 159 patients developed multiple organ metastases. Metastatic disease developed in 656 (7.12% of all patients with NET) patients with NET and 991 (30.20% of all patients with NEC) patients with NEC. The liver was the most common site of metastasis and comprised 74.80% (including solitary and multiple metastases) of all metastatic disease; furthermore, solitary liver metastases composed a high proportion of all liver metastases (87.17%). We separated patients into the NET group and NEC group and summarized the metastasis information (including specific solitary metastasis and two most frequent types of multiple metastasis) based on the primary sites in Figure 4. NEC obviously had a higher potential than NET to develop extrahepatic metastasis at each primary site. With the exception of appendicular NENs, the liver was the most frequent metastasis site of all GI‐NENs. NET in the stomach and appendix only metastasized to the liver. Metastasis of NET in the esophagus was not observed, whereas esophageal NEC often metastasized to the liver (35.06%), including solitary liver metastases (55.56%) and liver‐based multiple organ metastases (44.44%). A total of 76.47% of stomach NENs metastasized to the liver, but solitary lung, brain, and bone metastases were not observed. NENs in the duodenum, jejunum, and ileum frequently metastasized to unspecific sites. The metastasis patterns of NET and NEC in small intestine were similar. Metastatic appendicular NENs were rare. Although liver metastasis comprised only 37.5% of all metastatic NECs, multiple organ metastases were not observed in this subtype. Approximately 80% of colonic NENs metastasized to the liver, among which 78.57% were solitary liver metastases. Concomitant liver and lung metastasis were the most common type of multiple metastases at the colon in both the NET and NEC groups. A total of 81.95% of rectal NENs metastasized to the liver, among which 67.78% were solitary liver metastases. Concomitant liver and bone metastasis were the most common type of multiple metastases at the rectum in both the NET and NEC groups.

### Exploration of risk factors for metastasis based on different primary sites

We performed univariate and multivariate logistic regressions to further explore the risk factors for the different primary sites in developing metastatic disease and summarized the results in Table S1. NEC definitely increases the risk of metastasis compared with NET at every primary site except the esophagus, and none of the factors included was an independent risk factor for esophageal NENs. For duodenal NENs, tumor sizes greater than 5 cm, depth of tumor invasion (>T3), and histology type were independent risk factor for metastasis. Grade IV tumor and N+ status were risk factors of metastatic jejunal and ileal NENs; additionally, these NENs had an increased risk of metastasis as the tumor size and the depth of tumor invasion increased. Age older than 60, tumor size greater than 5 cm, and grade IV disease were independent risk factors for appendicular NEN metastasis. For colonic NENs, positive regional lymph nodes, grade IV status, and pathological type were independent risk factors. Finally, gender, insurance status, tumor size, positive regional lymph nodes, and pathological type were independent risk factors for rectal NEN metastasis.

## Discussion

In our study, 13.17% of patients diagnosed GI‐NENs between 2010 and 2014 presented metastatic disease; this percentage was similar to the incidence observed using previous SEER data from 1973 to 1999 (12.9%) 9, 10 but was lower than the incidence observed using data from the Swedish Cancer Registry (23%) 11 and National Cancer Registry of Spain (44%) 12. Another study using a Canadian database showed that the proportion of individuals with metastases at presentation decreased from 1994 to 2009 (from 29% to 13%) 13. One possible reason for this change is that our study focused on GI‐NENs, while other studies that use the SEER database include all types of NENs. Another reason may be that more attention has been paid to this disease, and highly sensitive imaging techniques, including SPECT with ^111^In‐pentetreotide 14; positron emission tomography with ^68^Ga‐DOTATATE 15, ^11^C 5‐HTP 16, and ^18^F‐DOPA 17; and endoscopic ultrasound and video capsule endoscopy, are now available to help detect NENs at earlier disease stages. However, we still lack adequate epidemiological information, and studies of risk factors have focused on the clinical characteristics of metastatic GI‐NENs. As is commonly known, disease progression significantly impacts both the affected individual and their caregivers; this progression leads to a deteriorating quality of life as well as consumption of healthcare resources. Therefore, it is important to identify individuals at higher risk of developing metastatic disease and implement different follow‐up strategies for these individuals. Therefore, we used a population‐based approach to analyze the epidemiologic characteristics of metastatic GI‐NENs and to explore the risk factors of metastases that arise from various primary sites.

We know that NENs are a heterogeneous disease arising from different cells distributed in various organs and tissues 18. Therefore, we divided the patients by their primary sites before conducting further analyses. The results revealed that the primary sites are important regarding the metastatic behavior. We observed different metastatic patterns and metastasis‐related risk factors among the different primary sites. However, no studies based on population have analyzed GI‐NENs by anatomic origin. Further evidence of clinical characteristics associated with the difference of survival among the different sites is insufficient. What is more, there are limited studies with primary site‐specific data; these analyses would be meaningful to clinical physicians to easily identify high‐risk patients.

Recently, accumulating research has suggested that NEC and NET are different, especially at the gene level. Tang and his colleagues examined 33 cases of pancreatic G3 neuroendocrine neoplasms and found that G3 NETs were different than NEC in the expression and mutation of genes, suggesting that NECs are similar to adenocarcinoma in the pancreas 19. Takizawa et al. 20 examined 25 colorectal NECs and concluded that the molecular features of colorectal NECs are similar to those of adenocarcinomas, not NETs, and hypothesized that Rb‐p16 pathway disruption may contribute to the promotion of proliferative activity in colorectal NECs. Additionally, ENETS updated the guidelines separating WHO G3 into G3 NET and G3 NEC, which stated that G3 NET and G3 NEC showed different characteristics and prognoses 1. Therefore, it is important and rational to separate patients into NET and NEC groups for further analysis. Our study separated patients into these two groups and further investigated the survival of metastatic disease at each primary site. The results suggested that the differences in survival of metastatic NET among different primary sites were not as significant as in NEC. We then explored the metastasis pattern of NET and NEC at each primary site, which revealed that NEC had a higher potential than NET to develop extrahepatic metastasis at all primary sites. We call for more research focused on these differences, which will be indispensable for establishing guidelines in the future.

Our study performed a Cox regression to identify risk factors of OS and logistic regression to determine the risk factors for metastasis in the different primary sites. Our data show that esophageal NENs have the worst survival, which is a blind area of GI‐NENs. Additionally, the results of the Cox analysis (using the esophagus as a reference and adjusting for the influence of gender, race, and marital status) revealed that other primary sites except the colon and stomach could be protective factors of OS, which is consistent with the results of the survival analysis. Further analysis may partially explain the result that esophageal NENs have the highest rate of metastasis and that the metastasis pattern of esophageal NENs showed high a probability to develop multi‐organ metastases. We urge more attention to esophageal NENs, which are still treated the same way as esophageal cancer. Another interesting point we observed in this study is that previous studies often treat duodenal, jejunal, and ileal NENs as the same disease 21. We subcategorized small intestine NENs into duodenal, jejunal, and ileal NENs and conducted further analysis; the results of which suggested that these subtypes are different in many aspects. Jejunal and ileal NENs are more likely to metastasize than duodenal NENs (26.87% vs. 18.30%, *P *<* *0.05). They also have a different metastasis pattern, as jejunal and ileal NENs have a higher rate of distant lymph nodes metastasis and multi‐organ metastasis. The above data may explain the better 4‐year OS of duodenal NENs compared with jejunal and ileal NENs. The risk factors for metastases are also different among these subtypes. Larger tumor size will increase the risk of metastasis in jejunal and ileal NENs, but no specific relevant factors were observed in duodenal NENs. Therefore, we suppose that small intestine NENs should be divided into duodenal and jejunal/ileal subtypes, which have different embryonic origin and showed different biological behaviors in our study. This conclusion is consistent with some retrospective studies 22, 23, 24. The risk factors for metastasis presented in our study suggested that tumor grade, histology, T‐stage, and N‐stage do not affect the metastatic status in some primary sites, such as the esophagus and stomach. Indeed, NETs currently lack a unified specific staging system beyond the WHO classification. Therefore, we need to establish a more powerful predictive system for the different primary sites to evaluate patients and identify individuals at increased risk of developing metastatic disease to provide radical and timely treatment.

We also summarized the probability of metastasis sites and found that NENs show an apparent preference for liver metastasis. This phenomenon has been suggested by other previous studies 6. Solitary metastasis is common in liver metastases; by contrast, lung, bone, and brain metastases presented much higher rates of multiple metastases, which have not been demonstrated to date. In our study, more than 70% of patients who developed lung, bone, or brain metastases presented multiple metastatic lesions. As is commonly known, the liver is considered an immunosuppressive organ. Although tumors have an increased tendency to thrive in the liver, they are antigenic for other organs 25. This phenomenon might also partially explain why solitary liver metastases were more common than other metastasis sites. This result also provides important information for physicians to establish a proper pretreatment evaluation of patients. For example, a patient diagnosed with NEN by biopsy from a metastasis site such as the lung will likely undergo more imaging examinations such as whole‐body bone scanning or liver nuclear magnetic resonance. These observations also remind our physicians that patients with metastatic disease should be monitored closely and provide more evidence to physicians to make better informed decisions regarding the patients’ follow‐up strategy.

To the best of our knowledge, this is the first time that the specific metastasis patterns, metastatic‐related survival analyses, and risk factors of metastatic disease of GI‐NETs and GI‐NECs have been assessed at the population level using the SEER database. However, there are several limitations to this study. First, due to the absence of chemotherapy‐related information in the SEER database, the effects of chemotherapy on survival could not be evaluated. Second, in the SEER database, only metastases to the following sites were included: bone, lung, liver, brain, and distant lymph node. Although these five sites are common distant sites for metastasis in GI‐NENs, the present study revealed that subdividing GI‐NENs into different primary sites resulted in classifying 50% of metastasis sites from appendicular NEN patients as nonspecific. Third, the SEER database only included synchronous metastasis information. Some patients who might have developed metachronous metastatic lesions were not included in our study, which might lead to an underestimation of extrahepatic metastasis.

## Conclusion

GI‐NENs are a group of heterogeneous diseases that show apparent differences in specific metastasis pattern, OS, and risk factors for metastatic disease depending on the primary site. Esophageal NENs showed the worst survival, and NENs in the small intestine should be divided into duodenal NENs and jejunal and ileal NENs. Physicians can decide the follow‐up strategy for each primary site by referring to the survival data and metastasis patterns presented here. GI‐NETs and GI‐NECs exhibited differences in survival when developing metastatic disease. The metastasis pattern suggested that NEC had a higher potential to develop extrahepatic metastasis at all primary sites than did NET. Future studies of GI‐NENs should be based on different primary sites and separate patients into GI‐NET and GI‐NEC groups to guide the pretreatment evaluation of patients and allow more rational follow‐up strategies. More studies focused on the clinicopathological differences between NET and NEC, and the establishment of a more powerful predictive system for different primary sites is warranted to identify high‐risk patients and predict survival.

## Conflict of Interest

The authors declare no conflict of interest.

## Supporting information


**Table S1.** Univariate and multivariate analyses of risk factors of the different primary sites in developing metastatic disease.
