# Supplementary figures and images for: Pattern and risk factors for distant metastases in gastrointestinal neuroendocrine neoplasms: a population‐based study
Source: Cancer Med. 2018 May 7;7(6):2699–709. doi: 10.1002/cam4.1507 (PMC6010810; doi:10.1002/cam4.1507)

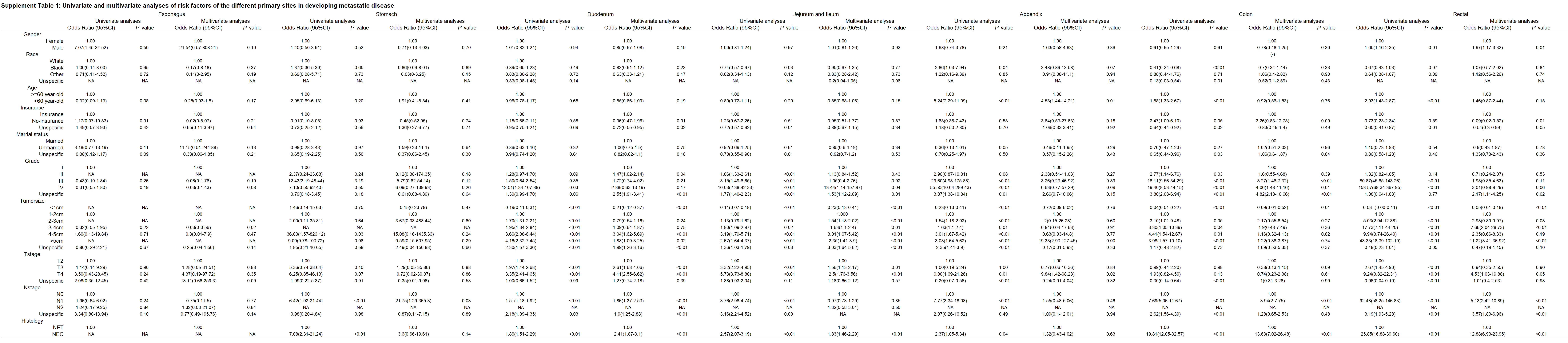

Supplement: Supplementary file 2 [file CAM4-7-2699-s002.tif]
